# Differentiation of myomas by means of biomagnetic and doppler findings

**DOI:** 10.1186/1477-044X-4-3

**Published:** 2006-04-03

**Authors:** Panagiotis Anastasiadis, Achilleas N Anastasiadis, Athanasia Kotini, Nikoleta Koutlaki, Photios Anninos

**Affiliations:** 1 Department of Obstetrics and Gynecology, Medical School, Democritus University of Thrace, University Campus, Alexandroupolis, 68100, Greece; 2 Laboratory of Medical Physics, Medical School Democritus University of Thrace, University Campus, Alexandroupolis, 68100, Greece

## Abstract

**Aim:**

To elucidate the hemodynamics of the uterine artery myomas by use of Doppler ultrasound and biomagnetic measurements.

**Method:**

Twenty-four women were included in the study. Sixteen of them were characterised with large myomas whereas 8 of them with small ones. Biomagnetic signals of uterine arteries myomas were recorded and analyzed with Fourier analysis. The biomagnetic signals were distributed according to spectral amplitudes as high (140–300 ft/√Hz), low (50–110 ft/√Hz) and borderline (111–139 ft/√Hz). Uterine artery waveform measurements were evaluated by use of Pulsatility Index (PI) (normal value PI < 1.45).

**Results:**

There was a statistically significant difference between large and small myomas concerning the waveform amplitudes (P < 0.0005) and the PI index (P < 0.0005). Specifically, we noticed high biomagnetic amplitudes in most large myomas (93.75 %) and low biomagnetic amplitudes in most small ones (87.5 %).

**Conclusion:**

It is suggested that the biomagnetic recordings of uterine artery myomas could be a valuable modality in the estimation of the circulation of blood cells justifying the findings of Doppler velocimetry examination.

## Introduction

Uterine myomas irrespective of whether they are small and asymptomatic (as in the postmenopausal women) or large and symptomatic (as in premenopausal women) considerably affect uterine artery blood flow velocity. Benign uterine leiomyomas are usually easily recognized with gray-scale ultrasonography, but may sometimes be difficult to differentiate from solid ovarian tumours. Doppler ultrasound is a diagnostic modality widely applied in obstetrics and gynecology [1-5]. It has been used to characterize pelvic tumours, and transvaginal color and spectral Doppler examinations have been suggested to enable discrimination between benign and malignant adnexal masses [6-8]. Circulation of benign uterine leimyomas has been described by use of Doppler velocimetry. Some authors have tried to correlate the myoma volume and the blood flow circulation in the arteries of the wall of the myomas [9]. Much higher blood flow velocities were recorded in the arteries of large myomas than in small myomas [10]. PI values<1 in a pelvic mass have been taken to indicate malignancy. However Kurjak *et al*. [11] reported the arteries of many myomas to manifest low blood flow impedance and high velocity blood flow. PI values<1 are common in uterine myomas and do not indicate malignancy. Uterine artery blood flow velocity reflects uterine perfusion, and low uterine artery PI values might originate from the need for increased blood supply in uteri with large myomas as a consequence of the increased uterine volume.

The myomas lesion, like any other living tissue, emits spontaneous magnetic field caused by ionic movements across the plasma membrane [12-15]. This activity, although exceedingly week (it is about 10^-8 ^of the earth's magnetic field which is equivalent to 50 μT) can be measured by means of a superconducting quantum interference device (SQUID). In recent years SQUID biomagnetometry has proven to be most helpful in the study of hemodynamics of certain vessels by measuring the exceedingly weak magnetic fields emitted by circulating blood cells. The higher the concentration of blood cells in the tested area, the higher the biomagnetic fields produced and the higher the recorded measurements. This technique has been successfully used for studying fetal heart, brain activity, hemodynamics of the umbilical artery and more recently for the investigation of breast and ovarian tumors [12-19].

Our study aims to report the characteristics of the biomagnetic recordings obtained from uteri with myomas and to correlate them with the corresponding Doppler values in order to test the validity of the biomagnetometer SQUID in the evaluation of the hemodynamics of the blood flow circulation of uterine myomas.

## Methods

The group study comprised 24 premenopausal women who were planed to undergo laparotomy because of symptomatic myomas. Sixteen of them were characterised with large myomas whereas 8 of them with small ones. The diagnosis of myomas was made by use of bimanual gynecologic examination as well as with both transabdominal and transvaginal gray-scale sonography. The transabdominal examination was necessary to adequately measure uterine size in women with large uterus. Transvaginal examination was performed with a woman in the lithotomy position. Myoma volume was expressed in cm3 and was calculated according to the formula length (cm) × depth (cm) × width (cm) × 0.5. A myoma was considered large if at least one of its diameters was > 5 cm; otherwise it was characterized a small one. If more than one myoma was found in the pelvis, the largest myoma was examined. The uterine arteries and myoma vascularization were visualized by the color Doppler technique. Blood flow velocity waveforms from both uterine arteries were obtained by placing the Doppler gate over the colour areas and activating the pulsed Doppler function. The main stem of the uterine arteries was examined lateral to cervix at the level of the internal os. The mean value from the PI obtained from the right and left uterine artery of each patient was recorded and correlated with the myoma volume and with the corresponding biomagnetic measurements. All women underwent hysterectomy or excision of the myoma and histologic diagnosis of a benign uterine myoma was made for all of them.

Biomagnetic recordings were obtained by a single channel second order gradiometer DC-SQUID (MODEL 601; Biomagnetic Technologies Inc., San Diego, USA) [12-18]. The gradiometer operates at low liquid helium temperature (4°K) with a sensitivity of 95 pTesla/Volt at 1000 Hz. To attenuate the influence of electromagnetic artifacts, the measurements were performed in a shielded room of low magnetic noise. Ultrasound scanner Doppler examination assessed prior to the procedure the exact placement of myomas in order to be sure that the biomagnetic signals from nearby vessels were excluded. During the recording procedure the patient was relaxed lying supine on a wooden bed free of any metallic object so as to decrease the environmental noise and get better signal to noise ratio. The recordings were performed after positioning the SQUID sensor 3 mm above the exact position of myomas assessed by the Doppler examination in order to allow the maximum magnetic flux to pass through the coil with little deviation from the vertical direction. None of the women were reluctant about the method and none of the volunteers withdrew from the study. Five points were selected for examination according to the myoma topography. Point 5 was located at the very center of the myoma, whereas points 1–4 were located at the periphery of the target area. For each point 32 recordings of 1-second duration each were taken and digitized by a 12 bit precision analogue-to-digital converter with a sampling frequency of 256 Hz. The duration of the above recordings is justified because the chosen time interval is enough to cancel out, on the average, all random events and to allow only persistent ones to remain. The biomagnetic signals were band-pass filtered, with cut-off-frequencies of 0.1–100 Hz. The associated Nyquist frequency limit, with the above-mentioned sampling frequency, is therefore 128 Hz, which is well above the constituent frequency components of interest in biomagnetic recordings and avoids aliasing artifacts. Using an analog-to-digital converter, the analog signals were converted to digital ones and after Fourier statistical analysis the average spectral densities from the 32 recordings of magnetic field strength were obtained from each one of the 5 points measured in the frequency range 2–7 Hz. Measurements of areas of interest (signals) where were related to measurements of background magnetic activity (noise) in all patients. By convention the maximum value was used when assessing the myomas. The obstetricians were ignorant of the biomagnetic values. Informed consent for the study was obtained from all the patients prior to the procedure. The biomagnetic signals were distributed according to spectral amplitudes as high (140–300 ft/√Hz), low (50–110 ft/√Hz) and borderline (111–139 ft/√Hz). Statistical analysis was obtained using t-test.

## Results

**Figure 1:**
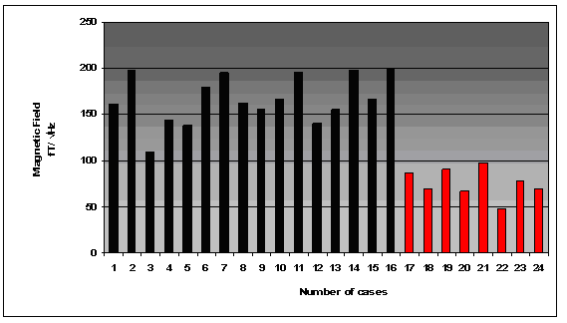
The spontaneous magnetic activity generated from the 24 women with myomas.

**Figure 2:**
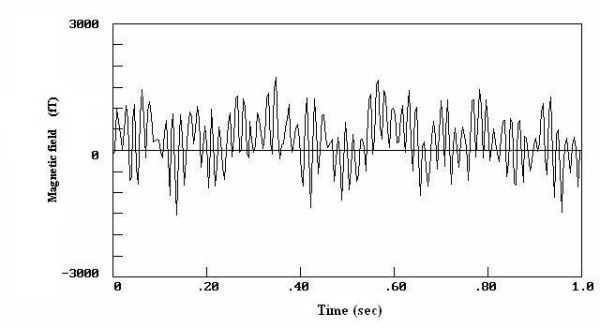
The wave-form of the magnetic field emitted from a woman with large myoma.

**Figure 3:**
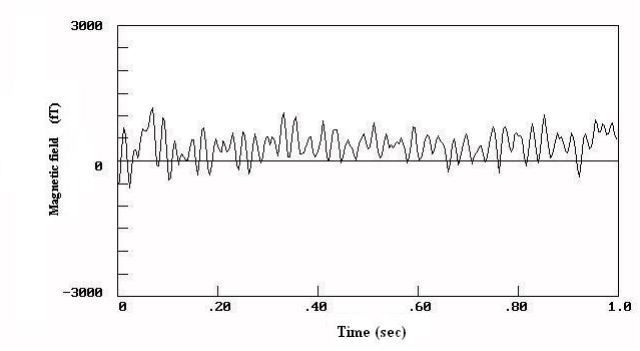
The wave-form of the magnetic field emitted from a woman with small myoma.

**Figure 4:**
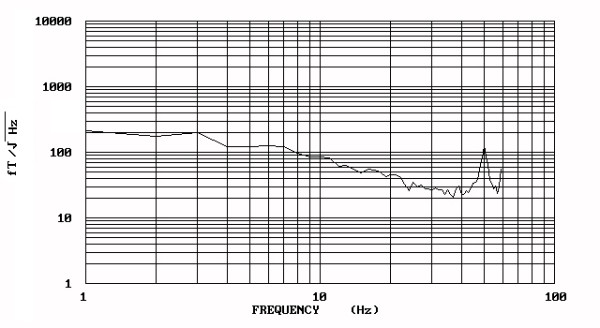
The spectral densities of the wave-form illustrated in fig. 2. High spectral amplitudes (198 fT/√Hz) are distributed in the frequency of 3 Hz.

**Figure 5:**
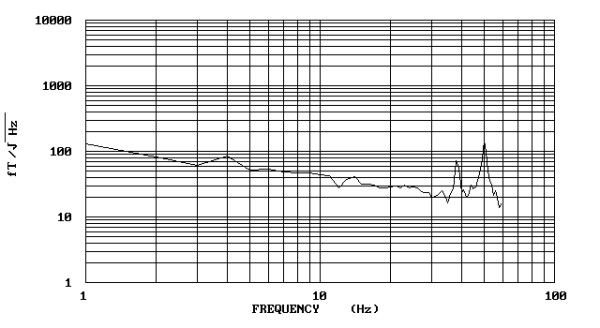
The spectral densities of the wave-form illustrated in fig. 3. Low spectral amplitudes (86 fT/√Hz) are distributed in the frequency of 4 Hz.

**Figure 6:**
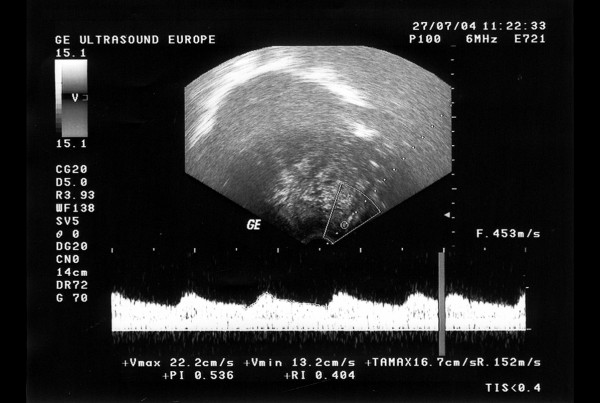
Typical Doppler image obtained from a uterine myoma.

The results for the biomagnetic data from the maximum value of the 5 measured points of each patient are indicated in Figure 1. The raw data were of high amplitudes in most (93.75 %) of the large uterine myomas (Fig. 2) and low amplitudes in most (87.5 %) of the small ones (Fig. 3). There was 1 case in the 1^st ^group with low biomagnetic values (one patient with firm pelvic adhesions due to previous peritonitis) and one case in the 2^nd ^group with very low values which corresponded to an intensively retroflective uterus. The corresponding spectral densities of the magnetic field were shown after statistical Fourier analysis: these were of high spectral amplitudes in the apparently large myomas (Fig. 4) and of low spectral amplitudes in small ones (Fig. 5). The spectral peaks at 50 Hz referred to the power supply (Figs 4, 5). The maximum total average of spectral amplitudes emitted by the large myomas was: 166.68 ± 26.35 fT/√Hz whereas in the small ones 75.62 ± 15.79 fT/√Hz. There was no overlap in the mean values of the two study groups. The above difference was of statistical significance (P < 0.0005). Typical Doppler image obtained from a uterine myoma is shown in Figure 6.

Table 1 presents the mean values of the myoma volume, the uterine artery PI and the biomagnetic amplitudes in the two study groups. A statistically significant difference was observed in the PI values obtained from large and small myomas respectively (P < 0.0005). Higher PI values were recorded in the uterine arteries of uteri with small myomas, while lower PI values were observed in the uterine arteries supplying large myomas.

**Table 1:** Mean values of myoma volume, uterine artery PI and biomagnetic amplitude in the two study groups

	**Volume**	**PI**	**Biomagnetic amplitude (fT/√Hz)**
**Large myomas**	287.19 Range (99.8–1119.4)	43.11 Range (29.6–56.25)	166.68 ± 26.35
**Small myomas**	1.06 Range (0.67–1.29)	2.02 Range (1.19–2.76)	75.62 ± 15.79
**P**	<0.0005	<0.0005	<0.0005

## Discussion

The data presented in this study, although preliminary, suggest a potential validity of biomagnetism in the differentiation of uterine myomas. This is not unexpected as malignant tissues, by virtue of their rapid expansion, vascularity and thus increased ionic movements produce magnetic fields of higher intensity than normal tissues [15,18,20].

It is well known that tumor hyperemia is related to new blood vessel growth (neovascularization) as well as to dilatation of previously existing vessels. Viable tumor cells release diffusible angiogenic factors, which stimulate new capillary growth and endothelial mitosis in vivo [21] even when tumor cell proliferation has been arrested by irradiation [22]. Folkman *et al*. [23] proposed a hypothesis that "once tumor take occurs", every further increase in tumor cell population must be preceded by an increase in new capillaries which converge upon the tumor in early growth. According to this concept, a small focus of tumor cells could not increase indefinitely without the induction of angiogenesis. Furthermore, there is strong evidence that growth of solid tumors beyond a few millimeters in diameter depends on the induction of functional microcirculation from the surrounding host tissue. It is obvious that malignant tumor induces growth of the independent and characteristic vascular network on its own. The tumor vasculature is highly heterogeneous and does not conform to standard normal vascular organization (i.e. artery, to arteriole, to capillaries, to postcapillary venule, to venule, to vein). A key difference between normal and tumor vessels is that the latter are dilated, saccular and tortuous, and may contain tumor cells within the endothelial lining of the vessel wall [24].

It has been well established in previous studies that low uterine artery PI values are present in uteri with myomas. This may be an effect of increased uterine size and not necessarily an effect of the myomas per se. Differences in the uterine artery PI values might also reflect differences in the women's menstrual status (pre-menopausal vs menopausal women). In this study all women were pre-menopausal and the difference in PI values between the two groups is more likely to be explained by the difference in the myoma size.

Doppler velocimetry studies on myoma vessels (both capsule and core vessels) have shown that PI values < 1.0 are common in uterine myomas and do not indicate malignancy. This eliminates the role of Doppler study in the discrimination of benign uterine myomas from other malignant pelvic tumors. Biomagnetic recordings obtained from benign and malignant tumors of various organs (eg breast, ovary) proved to be helpful in the differentiation of the tumor's biologic behavior [12-18]. Further biomagnetic studies on uterine myomas might elucidate the value of biomagnetism in the differential diagnosis of benign and malignant pelvic tumors.

The data presented in the study, although preliminary, justify a novel approach to biomagnetism and suggest that this imaging modality of measuring the magnetic activity of uterine artery circulation can be potentially exploited in follow up of women with myomas. It is a non-invasive procedure, reliable, rapid and easy to interpret. Furthermore, it is totally harmless and well tolerated by women. It is true that SQUID biomagnetometry needs special equipment, a suitable prepared room and a good methodological knowledge, but once these requirements have been met, the method is rewarding. A further refinement of the equipment so as to be more sensitive and easily moved to the patients' bedside could be most helpful in clinical practice. Nevertheless, further studies in large numbers of women are needed in order to evaluate the role of biomagnetometry in the differentiation of pelvic tumors and to investigate its potential role as an adjunct procedure to established diagnostic methods.
